# 

**Published:** 1909-10-09

**Authors:** 


### The Question Box.

**Special Notice.**—Questions of interest affecting the honorary medical staff and hospital administration in all departments are invited. When any point arises we hope our readers will formulate it in a question and so co-operate to make this column of real value and interest. In this way the experience of the best-managed hospitals should be made helpful to the whole hospital world. We shall welcome the contribution of both questions and answers.

**N.B.**—The publication of an answer in this column does not necessarily preclude the publication of subsequent answers to the same question, for a question may involve points which require to be threshed out and fully discussed. Answers, if published, will be paid for at scale rates.

### POOR LAW INFIRMARIES.

Question 1. Should Poor Law infirmaries have a visiting medical staff? If so, on what terms and conditions of attendance?

### ANSWERS.

#### A London View.

By Mr. John R. Lunn, F.R.C.S., Resident Medical Superintendent the Marylebone Infirmary.

I do not support any idea of a visiting staff to the infirmaries. I will bring your letter before our Medical Superintendents Society when we next meet.

#### A Provincial View.

By Mr. Jordan Lloyd, M.D., F.R.C.S., J.P., Visiting Surgeon Birmingham Poor Law Infirmary.

My answer to this question must necessarily be based on my own personal experience as visiting surgeon to the Birmingham Workhouse Infirmary during the past twenty years and more as I have little or no personal knowledge of the work of other Poor Law infirmaries. I should like to say here at the outset that the status of the visiting medical officer should be a high one—he should be in practice as a consultant in the case of large towns or cities, and in the smaller towns should be a recognised leader among the practitioners of his district, if not actually a consultant. With such presumably efficient visiting officers the medical work in the infirmaries would be kept up to a high and a modern standard, in my opinion. The work would be carried out on the lines of the larger general hospitals, and would almost necessarily therefore be well abreast the times. My own work in the infirmary at Birmingham has been carried on exactly in the same manner as at the Clinical Hospital (Queen's) to which I am attached, and so far as I can see the results have been equally good and the cost per patient to the ratepayers has been very materially less—more than two-thirds! I have usually been able to find good resident officers to serve under me, and should have found better perhaps had the salaries been higher—as I think they ought to be. I have never had any difficulties whatever with the lay officers or authorities.

*Terms and Conditions of Attendance.*

The visiting officers should be paid a salary proportioned to the size of the infirmary. Their appointments should be held under the central governing body (Local Government Board), but should be made by the local governing body (Guardians). The appointments should terminate at a certain age, say 50 years, or possibly later. They should be entitled to four weeks' holiday per annum—during which period the authorities provide for their substitute. Their compulsory services should be absolutely confined to attendance upon *bonâ fide* Poor Law patients, and should not extend to "officers" nor to cases of accident in "non-pauper" patients brought into the infirmary from without. They should visit the infirmary where their services are required, but never less than twice weekly. They should not be compelled to spend at least a fixed number of hours weekly or daily inside the infirmary. They should have the privilege of making post-mortem examinations of all patients dying whilst under their care, and such records should be officially kept.

### THE UNIFORM SYSTEM OF ACCOUNTS: RESERVE FUNDS.

Question 1.—Should ordinary repairs be treated in the accounts under the head of "Ordinary" or "Extraordinary" expenditure?

### ANSWERS.

#### A London View.

By Mr. Thos. Ryan, Secretary St. Mary's Hospital.

Under the old conditions it was a very convenient thing to class expensive repairs occurring at long intervals as "Extraordinary Expenditure." Of course, in principle it was incorrect, but in practice it was most convenient, because, as "Extraordinary Expenditure" was excluded from the figure on which the cost per occupied bed was computed, the classification of such repairs under it prevented sudden considerable fluctuations in the cost per occupied bed, which was a very desirable thing to do. But there is little doubt that some made a much freer use of the receptacle called "Extraordinary Expenditure" than others could bring themselves to do, and on the whole, I think, it must be admitted that it was a good thing when it was done away with altogether.

Now, of course, the position is quite clear. Every expenditure that is not capital expenditure must be included in the "Income and Expenditure Account." There every repair, whether it occurs frequently in one year, or only once in seven years, as the painting of interior walls, has to go. The system affects everyone alike, and there can be no heartburnings.

#### A Provincial View.

By Mr. Harry Johnson, House Governor and Secretary Leicester Infirmary.

This is a question which has for a very long time been a source of much careful consideration and inquiry upon the part of hospital committees, and the accounts of the various hospitals in the past reveal one indisputable fact—that hospital committees have preferred to continue old customs and methods of dealing with ordinary repairs to thinking out and carrying into execution a distinct plan in accord with the business methods now being adopted in hospital management. The definite lead taken by King Edward's Fund on the recommendation of the Committee of Secretaries has been of very great help to hospital committees and secretaries generally, and the adoption of the Fund's recommendation to delete extensive ordinary repairs from the heading "Extraordinary Expenditure" and treat them under the head of "Establishment Charges, Renewals and Repairs" not only has much to commend it, but its adoption will do a great deal to make the cost of upkeep of the different hospitals comparable.

What has hitherto been the custom? Zealous officials, anxious to make their accounts and their institution stand well in the eyes of the public, have welcomed the haven afforded by the heading "Extraordinary Expenditure" to group under it all and sundry expenses which could not be said wholly to represent expenditure for the year. Thus the addition of new plant and improvements in the equipment of every department, the cost of periodical painting and decoration, internal and external, renewals and repairs for maintaining the institution in a state of efficiency, and many similar items have generally been excluded from the cost of maintenance and regarded as "Extraordinary Expenditure"—the result being that the cost of maintenance has been lessened, and the institution has thereby been able to show not only a low cost per head per patient, but to claim a reputation for efficient administration which it is doubtful if the figures have merited.

But is this the final solution of an important question? I think not. As The Hospital has so aptly remarked in Sir Henry Burdett's Report of the North Staffordshire Infirmary (issue of October 2, p. 17) "to be an efficient member of an hospital board entails labour and responsibility upon the individual, but every man who consents to join a hospital committee should be prepared to render personal service . . . to the full extent of the requirements of any hospital." An efficient board of men experienced in divers business matters is a *sine quâ non* to the good management of any hospital, and it is inconceivable that a plan universally adopted in all well-managed businesses, of making provision each year for future extensions or enlarged activities by allocating to a reserve fund a part of the profits of each year, can long be delayed in hospital administration. It may be replied that the hospitals are not in the position of business concerns trading for profit, that it seldom happens that the income of hospitals shows a margin over expenditure, and that it would be impossible to form a reserve fund. But do not many businesses build up a reserve fund, even when profits are small, at the expense of the profit sharers? And have not unsuccessful businesses been known to allocate sums to reserve at the cost of additions to the deficiency of the year? And if this method of finance finds approval with experienced business men in commercial life, can it possibly be bad finance in hospital life? At any rate trial will be made, and the public will be able to test the result of such a plan shortly. The Governors of the Leicester Infirmary propose at the close of this year to form a reserve fund to meet expenses of an exceptional nature, such as external decorations. This will be done notwithstanding the fact that a deficiency of £2,726 was brought forward to 1909 account. It is purposed to continue to add a sum each year to reserve, and when large expenditure is called for, to transfer an amount from the reserve fund to the "General Income Account" for the year. This reserve fund will be available for any and every expenditure of an exceptional nature. The board have no doubt of the success of their plan, even though its adoption may mean a temporary addition to the "Overdraft at the Bank." The Hospital "Question Box" provides a means for an expression of opinion on the part of hospital administrators as to the wisdom of making provision for ordinary repairs in the manner indicated. The suggested plan is regarded as one of importance to hospitals generally.

The Governors of the Leicester Infirmary would welcome a thorough discussion of their attempt to deal with the question.

### THE GRADUATES' MEDICAL SCHOOLS.

DIARY FOR THE WEEK, OCTOBER 11 to 16.

**MEDICAL GRADUATES' COLLEGE AND POLYCLINIC, 22 Chenies Street, W.C.**

**Clinique.**

**At 4 p.m.**

**October 11,** Dr. S. E. Dore, **Skin.**

**At 5.15 p.m.**
**October 11,** Dr. Harry Campbell, **Treatment of Neuralgia.**

**At 4 p.m.**
**October 12,** Dr. Essex Wynter, **Medical.**

**At 5.15 p.m.**
**October 12,** Dr. E. C. Hort, **Rational Immunisation from a Practical Standpoint.**

**At 4 p.m.**
**October 13,** Mr. Thomson Walker, **Surgical.**

**At 5.15 p.m.**
**October 13,** Dr. G. H. Savage, **Moral Insanity.**

**At 4 p.m.**
**October 14,** Sir Jonathan Hutchinson, **Surgical.**

**At 5.15 p.m.**
**October 14,** Mr. Lockhart Mummery, **The Diagnosis and Treatment of the more serious forms of Colitis.**

**At 4 p.m.**
**October 15,** Mr. W. Stuart-Low, **Ear, Nose, and Throat.**

The "Medical Directory for 1910," published by Messrs. Churchill, 7 Great Marlborough Street, London, W., will contain a descriptive list of all the most important British spas and health resorts, compiled by Dr. Norman Hay Forbes, ex-Vice-President of the British Balneological and Climatological Society. The Directory is now being compiled, but the editors can still receive information. The same firm published last week another revised edition of Dr. Hale White's "Manual of Materia Medica." This work was first published in 1892. Its great popularity is evidenced by the fact that an eleventh edition is now called for.

